# Geographically weighted negative binomial regression for spatial count data: Methodology and application

**DOI:** 10.1016/j.mex.2026.103919

**Published:** 2026-04-19

**Authors:** Toha Saifudin, Nur Chamidah, Nur Azizah, Fayza Shafira Renianti, Nashwa Carista, Irsyad Yoga Adyatma, Izhar Muhammad Tianda

**Affiliations:** Study Program of Statistics, Department of Mathematics, Faculty of Sciences and Technology, Airlangga University, Surabaya, Indonesia

**Keywords:** Geographically weighted negative binomial regression, Negative binomial regression, Poisson regression, Count data, Spatial heterogeneity, Overdispersion, HIV

## Abstract

This study presents a reproducible methodological framework of Geographically Weighted Negative Binomial Regression (GWNBR) for modeling spatially overdispersed count data. The framework integrates Negative Binomial Regression (NBR) model with spatial weighting to capture overdispersion and spatial heterogeneity. The modeling procedure begins with Poisson regression, followed by an overdispersion test to justify the use of NBR. Spatial dependence and heterogeneity are then evaluated using Moran’s I and Breusch-Pagan diagnostics. The GWNBR model is subsequently constructed using kernel-based spatial weighting with bandwidth selection through cross-validation. Model performance is assessed using Akaike Information Criterion (AIC) to compare Poisson, NBR, and GWNBR. A real-data application involving HIV case in Indonesia illustrates the capability of GWNBR to produce spatially localized estimates, offering enhanced interpretability and model accuracy over global approaches. This study contributes transparent and step-by-step analytical workflow that enhances reproducibility in spatial count data modeling.

## The highlights of this research are

• Count data modeling was initiated using Poisson regression, and overdispersion was tested to justify the use of NBR.
• Spatial dependence and heterogeneity were tested using Moran’s I and Breusch-Pagan tests, followed by localized modeling with GWNBR.
• Model performance was compared using AIC to evaluate improvements from global to spatially weighted count models.

## Graphical abstract

**Figure:**
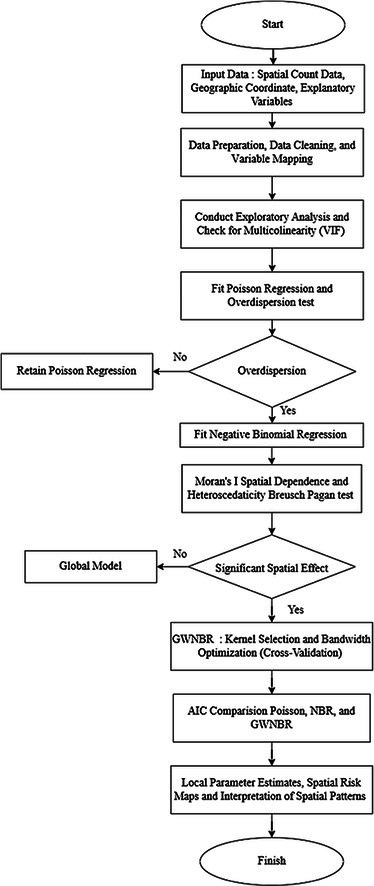

**Specifications table**

**Table:** 

**Subject area**	Mathematics and Statistics
**More specific subject area**	Spatial Statistics and Count Data Modeling
**Name of your method**	Geographically Weighted Negative Binomial Regression
**Name and reference of original method**	Negative Binomial Regression by Hilbe (2011), J. M. Hilbe, Negative Binomial Regression (2nd ed.)., Cambridge University Press, 2011, https://doi.org/10.1017/CBO9780511973420. Geographically Weighted Regression by Fotheringham et al. (2002), A. S. Fotheringham, C. Brunsdon, and M. Charlton, Geographically Weighted Regression: The Analysis of Spatially Varying Relationships, John Wiley & Sons, 2002.
Resource availability	HIV case data and explanatory variables (poverty rate, education, village status, and expenditure) are available from the Ministry of Health of Indonesia and the Central Bureau of Statistics (BPS) as follows: https://www.kemkes.go.id https://www.bps.go.id

## Background

Human Immunodeficiency Virus (HIV) remains a significant public health issue in Indonesia. It is a chronic infectious disease transmitted primarily through contact with infected bodily fluids such as blood, semen, and vaginal secretions, and can progress to Acquired Immunodeficiency Syndrome (AIDS) if untreated [1,2]. According to data from the Indonesian Ministry of Health in 2023, Indonesia recorded 57,299 new HIV cases, the second highest in Asia with the highest incidence observed in East Java, West Java, Jakarta, and Central Java [3]. This increasing trend underscores the urgency of addressing HIV within the framework of Sustainable Development Goals (SDGs) 3, particularly target 3.3 which aims to end the AIDS epidemic by 2030. The uneven distribution of cases across regions reflects spatial disparities driven by socioeconomic and geographic factors [4].

Regression modeling is a widely used approach to explain relationships between explanatory variables and a response variable. For count data such as HIV cases, Poisson regression is commonly applied under the assumption that the mean and variance are equal [5]. However, real-world data frequently violate this assumption, leading to overdispersion, which can bias parameter estimates. The Negative Binomial Regression (NBR) model addresses this issue by introducing a dispersion parameter to accommodate the extra-Poisson variability [6]. Yet, conventional global models like NBR assume spatial stationarity that the relationships between variables are constant across locations. This assumption is problematic in s patial epidemiological contexts where regional characteristics vary. To examine spatial dependence and heteroskedasticity tests such as Moran’s I and the Breusch-Pagan are commonly employed [7,8].

Geographically Weighted Negative Binomial Regression (GWNBR) extends the NBR framework by incorporating spatial weighting functions, allowing model coefficients to vary across geographic locations [9]. This enables the model to account simultaneously for overdispersion and spatial heterogeneity. Kernel functions, such as Gaussian and Bisquare, are used to define spatial weights, while optimal bandwidth selection is typically conducted via Cross-Validation (CV) [10]. GWNBR has been successfully applied in modeling spatial variation of diseases such as tuberculosis and dengue fever in Indonesia [11,12]. These studies demonstrate that local factors, such as healthcare access, sanitation, and population density may exert varying levels of influence across regions.

Although several previous studies have applied GWNBR in public health research, its use in modeling HIV cases in Indonesia remains limited. Previous studies either focus on global models or employ spatial regression without addressing overdispersion in count data. While GWNBR has gained traction in spatial epidemiology, its methodological application often lacks standardization, particularly in how overdispersion and spatial heterogeneity are handled simultaneously. Many studies apply models without fully integrating dispersion control and spatial adaptation. Moreover, few have systematically compared baseline count models with their spatial counterparts, which is essential for evaluating model suitability in overdispersed spatial data. This study introduces a structured and reproducible implementation of GWNBR, incorporating model comparison, kernel function evaluation, and bandwidth optimization via Cross-Validation. The proposed framework emphasizes model accuracy, interpretability of spatially varying coefficients, and reproducibility using open-source tools, providing a comprehensive approach for localized modeling of spatial count data.

## Methods details

### Poisson regression

Poisson regression is the fundamental statistical model for analyzing count data, where the response variable $Y_{i}$ represents the number of occurrences of an event within a fixed unit of time, space, or population [13]. The key assumption of the Poisson distribution is equidispersion, meaning that the conditional mean and variance of the response are equal $E\left(Y_{i}\right)=Var\left(Y_{i}\right)=\mu_{i}$. The probability mass function of the Poisson distribution is written in Eq. (1).

(1) $$\begin{array}{c} f\left(y_{i};\mu_{i}\right)=\frac{e^{-\mu_{i}}\mu_{i}^{y_{i}}}{y_{i}!},\;y_{i}=0,1,2,\cdots \end{array}$$

where $y_{i}$ denotes the observed count, $\mu_{i}>0$ denotes the expected number of events for observation i, and e represents Euler’s number [14]. To connect the mean count with explanatory variables, Poisson regression employs a log-link function in Eq. (2) below.

(2) $$\begin{array}{c} \log\left(\mu_{i}\right)=\beta_{0}+\beta_{1}x_{i1}+\ldots +\beta_{p}x_{ip} \end{array}$$

where $x_{ij}$ represents the j-th explanatory of observation i and $\beta_{0},\beta_{1},\ldots ,\beta_{p}$ are regression coefficients. In Poisson regression, the expected count is linked to a set of explanatory variables through a log-linear model as Eq. (3).

(3) $$\begin{array}{c} \mu_{i}=\exp\left(X_{i}\beta \right) \end{array}$$

Here, $X_{i}=\left(x_{i1},x_{i2},\ldots ,x_{ip}\right)$ represents the covariates and $\beta =(\beta_{0},\beta_{1},\ldots ,\beta_{p})$ denotes the parameter vector. This formulation ensures that the predicted counts are non-negative. Parameter estimation is performed using the Maximum Likelihood Estimation (MLE) method, which maximizes the probability of observing the data given the specified model [15]. The log-likelihood function of the Poisson regression model is written as Eq. (4).

(4) $$\begin{array}{c} l\left(\beta \right)=\sum_{i=1}^{n}\left(y_{i}\ln\left(\mu_{i}\right)-\mu_{i}-\ln\left(y_{i}!\right)\right) \end{array}$$

where n is the number of observations. The likelihood function encapsulates the contribution of each observation to the model, and maximizing it yields estimates of the regression parameters β.

Despite its popularity, the Poisson model often proves restrictive because of its equidispersion assumption. In practice, many datasets exhibit overdispersion $Var\left(Y_{i}\right)>E\left(Y_{i}\right)$ or underdispersion $Var\left(Y_{i}\right)<E\left(Y_{i}\right)$. Overdispersion leads to underestimation of standard errors and overly optimistic significance tests, which can produce biased statistical inference [16]. When overdispersion is present, the Negative Binomial Regression model provides a more flexible alternative by introducing an additional dispersion parameter that relaxes the equidispersion assumption.

### Negative binomial regression

In the Poisson regression model, the fundamental assumption is that the mean and the variance of the response variable are equal, expressed as $E\left(Y_{i}\right)=Var\left(Y_{i}\right)=\mu_{i}$. In many situations, this assumption does not hold, and the variance exceeds the mean. This phenomenon is referred to as overdispersion. When overdispersion occurs, the variance of the estimators becomes underestimated, which leads to incorrect inference. Therefore, it is necessary to evaluate whether the assumption of equidispersion is satisfied. The detection of overdispersion can be performed using the dispersion parameter θ, which is defined as the ratio between the deviance and the degrees of freedom written as Eq. (5).

(5) $$\begin{array}{c} \theta =\frac{D\left(\hat{\beta }\right)}{df} \end{array}$$

where df=n−p and p is the number of estimated parameters. The deviance statistic $D(\hat{\beta })$ is defined as Eq. (6) below.

(6) $$\begin{array}{c} D\left(\hat{\beta }\right)=-2\ln\Lambda =-2\ln\left(\frac{L\left(\omega \right)}{L\left(\Omega \right)}\right) \end{array}$$

where L(ω) is the likelihood of the fitted model and L(Ω) is the likelihood of the saturated model. When θ=0, the model reduces to the standard Poisson regression. A value of θ>0 captures overdispersion, while θ<0 corresponds to underdispersion [15].

To accommodate the presence of overdispersion, the Negative Binomial Regression (NBR) model can be applied. The Negative Binomial distribution is obtained as a Poisson-Gamma mixture distribution, in which the mean parameter of the Poisson distribution is assumed to follow a Gamma distribution. The probability mass function of the Negative Binomial distribution is given as Eq. (7).

(7) $$\begin{array}{c} f\left(y_{i};\mu_{i},\theta \right)=\frac{\Gamma \left(y_{i}+\frac{1}{\theta }\right)}{\Gamma \left(\frac{1}{\theta }\right)\Gamma \left(y_{i}+1\right)}{\left(\frac{1}{1+\theta \mu_{i}}\right)}^{\frac{1}{\theta }}{\left(\frac{\theta \mu_{i}}{1+\theta \mu_{i}}\right)}^{y_{i}} \end{array}$$

where Γ is the gamma function and θ>0 is the dispersion parameter [17]. The expectation and variance of the Negative Binomial distribution are $E\left(Y_{i}\right)=\mu_{i}$ and $Var\left(Y_{i}\right)=\mu_{i}+\theta \mu_{i}^{2}$. This expression shows that the variance is always greater than the mean whenever θ>0, thus making the model suitable when overdispersion exists in the data [18]. Furthermore, when θ=0, the variance reduces to $\mu_{i}$, which means the Negative Binomial distribution converges to the Poisson distribution [13].

The regression structure for the Negative Binomial model is similar to the Poisson regression, where the logarithm of the mean is expressed as a linear function of the covariates in Eq. (8).

(8) $$\begin{array}{c} \log\left(\mu_{i}\right)=\beta_{0}+\beta_{1}x_{i1}+\ldots +\beta_{p}x_{ip} \end{array}$$

The estimation of the parameters β and the dispersion parameter θ is carried out using the Maximum Likelihood Estimation (MLE) approach, where the log-likelihood function is written as Eq. (9).

(9) $$\begin{array}{c} l\left(\beta ,\theta \right)=\sum_{i=1}^{n}\left(\ln\Gamma \left(y_{i}+\frac{1}{\theta }\right)-\ln\Gamma \left(\frac{1}{\theta }\right)-\ln\Gamma \left(y_{i}+1\right)-\left(\frac{1}{\theta }\right)\ln\left(1+\theta \mu_{i}\right)+y_{i}\ln\left(\frac{\theta \mu_{i}}{1+\theta \mu_{i}}\right)\right) \end{array}$$

By including the dispersion parameter, the Negative Binomial regression is able to model count data with variance greater than the mean, which cannot be properly handled by the standard Poisson regression [16].

### Spatial weighting and geographically weighted negative binomial regression

Spatial regression is a statistical method used to analyze data that exhibits spatial dependence. Moran's I index is utilized to quantify spatial autocorrelation, defined as the extent to which a variable at a specific location exhibits a pattern of association with neighboring locations [6]. The index takes on values ranging from −1 to 1, with positive values denoting clustering (spatial aggregation) and negative values indicating a pattern of opposite dispersion (spatial dispersion). The Moran's I test statistic is calculated by the Eq. (10) below [19].

(10) $$\begin{array}{c} Z_{I}=\frac{I-E\left(I\right)}{\sqrt{var\left(I\right)}}\;with\;I=\frac{n}{\sum_{i}\sum_{j}w_{ij}}\times \frac{\sum_{i}\sum_{j}w_{ij}\left(y_{i}-\bar{y}\right)\left(y_{j}-\bar{y}\right)}{\sum_{i}{\left(y_{i}-\bar{y}\right)}^{2}} \end{array}$$

where $w_{ij}$ is the element of the i-th row and j-th column spatial weighting matrix, $y_{i}$ is the value of the response variable at location i, $y_{j}$ is the value of the response variable at location j, and $\bar{y}$ is the average of the response variable y. The hypothesis of Moran's I test is

$H_{0}:I=0$ (No spatial dependencies)

$H_{1}:I\neq 0$ (There is a spatial dependency)

The critical region of the test is that $H_{0}\;$is rejected if the value $|Z_{I}|>Z_{\alpha /2}$ or *p-value* < α. In this study, Moran’s I was applied to the residuals of the global Negative Binomial Regression model rather than raw counts to detect remaining spatial autocorrelation after accounting for covariates. Significant Moran’s I indicates unexplained spatial dependence, justifying the use of GWNBR. If Moran’s I index is statistically significant, it is necessary to employ a spatial regression approach for the purpose of capturing the effects of interregional linkages in the estimation model [20].

The Breusch-Pagan test is used to identify the presence of heteroscedasticity, which is a condition in which residual variants are not constant across regions. In the context of spatial regression, heteroscedasticity often arises due to differences in economic characteristics between regions [7]. The Breusch-Pagan test was applied to the Pearson residuals of the Negative Binomial Regression model to assess whether residual variance varies systematically across provinces. Using Pearson residuals rather than raw counts aligns the BP test with the generalized linear model (GLM) framework, as suggested in Hilbe [16]. The statistics of the Breusch-Pagan test are expressed in Eq. (11) as follows.

(11) $$\begin{array}{c} BP=\frac{1}{2}f^{T}{\left(Z^{T}Z\right)}^{-1}Z^{T}f \end{array}$$

where the element of vector f is $f_{i}=\left(\frac{e_{i}^{2}}{\sigma^{2}}-1\right)$, $e_{i}$ is the least square observation for the i-th observation. ***Z*** is an n×p sized matrix containing a vector of standardized covariates variables. The test hypothesis uses the Breusch-Pagan test statistics as follows.

$H_{0}:\sigma_{1}^{2}=\sigma_{2}^{2}=\ldots =\sigma_{n}^{2}=\sigma^{2}$ (Homoskedasticity)

$H_{1}$: there is at least one $\sigma_{i}^{2}\neq \sigma^{2};i=1,2,\ldots ,n$ (Heteroscedasticity)

The critical region is that $H_{0}$ is rejected if the value of $BP>\chi_{p}^{2}$ or *p-value* < α with p is number of covariates. In the event of heteroscedasticity being indicated by the test results, corrections must be implemented. Such corrections may take the form of robust estimation αp or the Generalized Least Squares (GLS) method, which serves to enhance the validity of the regression results [21]. By incorporating spatial autocorrelation analysis using Moran's Index I and conducting a heteroscedasticity test using the Breusch-Pagan test, the spatial regression approach can be a more valid and reliable analysis.

In regression analysis for spatial data, one of the fundamental assumptions is the independence of observations. However, in practice, observations located in nearby regions often exhibit spatial dependence. To address this, spatial econometric methods incorporate spatial weights that represent the geographical relationships among observational units [22]. Geographic coordinates (longitude and latitude) of provincial centroids were obtained from the official shapefiles provided by the Central Bureau of Statistics. These coordinates were used directly to compute Euclidean distances between provinces for constructing the spatial weight matrix. No coordinate projection or transformation was applied during the analysis.

A spatial weight matrix $W=[w_{ij}]$ is defined, where each element $w_{ij}$ reflects the spatial relationship between location i and location j. The weights are typically non-negative and satisfy $w_{ii}=0$. Spatial weights can be constructed using different approaches, including contiguity-based structures and distance-based structures. Contiguity-based weights assign $w_{ij}=1$ if region i and j share a common boundary and $w_{ij}=0$ otherwise, whereas distance-based matrices define $w_{ij}$ as a function of the geographical distance between i and j [23]. In this study, spatial weights were constructed using a distance-based kernel weighting scheme derived from Euclidean distances between provincial coordinates. This approach allows observations located closer in space to exert stronger influence than distant observations. The spatial weighting framework is incorporated into a geographically weighted modeling approach, allowing regression coefficients to vary across locations. The extension of regression to incorporate spatial heterogeneity can be achieved through Geographically Weighted Regression (GWR), which allows regression coefficients to vary across locations [24]. In this framework, each location i is associated with a set of coefficients $\beta (u_{i},v_{i})$, where $\left(u_{i},v_{i}\right)$ are the spatial coordinates of location i. The general form of the GWR model is expressed as Eq. (12).

(12) $$\begin{array}{c} y_{i}=\beta_{0}\left(u_{i},v_{i}\right)+\sum_{k=1}^{p}\beta_{k}\left(u_{i},v_{i}\right)x_{ik}+\varepsilon_{i} \end{array}$$

The parameters $\beta_{k}\left(u_{i},v_{i}\right)$ are estimated locally using weighted least squares, where the weights depend on the distance between observation i and the calibration point j. The local estimation of parameters is conducted through a kernel weighting function that assigns higher weights to observations closer to the focal location and lower weights to distant observations. A common choice is the adaptive bi-square kernel, defined as Eq. (13).

(13) $$\begin{array}{c} w_{\mathrm{ij}}\left(b_{i}\right)=\left\{ \begin{array}{c} {\left(1-{\left(\frac{d_{\mathrm{ij}}}{b_{i}}\right)}^{2}\right)}^{2},\;d_{\mathrm{ij}}<b_{i}\\ \;0,\;\mathrm{othe}\mathrm{rs} \end{array}\right. \end{array}$$

where $d_{ij}=\sqrt{{\left(u_{i},u_{j}\right)}^{2}+{\left(v_{i},v_{j}\right)}^{2}}$ is euclidian distance between location i and j, and $b_{i}$ is bandwidth at the i-th location [24]. In geographically weighted models, the bandwidth defines the spatial scale of the analysis. For adaptive kernels, it represents the proportion of nearest neighboring provinces used in local estimation, while for fixed kernels it corresponds to the distance between provincial centroids. Smaller bandwidths capture more localized spatial variation, whereas larger bandwidths produce smoother estimates over broader regions. To determine the optimal bandwidth, Cross-Validation (CV) is commonly used. The CV score is computed as Eq. (14).

(14) $$\begin{array}{c} CV\left(b\right)=\sum_{i=1}^{n}{\left(y_{i}-{\hat{y}}_{\neq i}\left(b\right)\right)}^{2} \end{array}$$

where ${\hat{y}}_{\neq i}\left(b\right)$ is the fitted value for i obtained by excluding i from the calibration and estimating the model with bandwidth b [24].

When the response variable is in the form of count data and overdispersion is present, the Geographically Weighted Negative Binomial Regression (GWNBR) model is applied [25]. This model combines the flexibility of the Negative Binomial regression in handling overdispersed count data with the spatial adaptivity of GWR. The mean structure of the GWNBR model is defined as Eq. (15).

(15) $$\begin{array}{c} \log\left(\mu_{i}\right)=\beta_{0}\left(u_{i},v_{i}\right)+\beta_{1}\left(u_{i},v_{i}\right)x_{i1}+\ldots +\beta_{p}\left(u_{i},v_{i}\right)x_{ip} \end{array}$$

The probability mass function of the Negative Binomial distribution is given as Eq. (16).

(16) $$\begin{array}{c} f\left(y_{i}|\beta_{j}\left(u_{i},v_{i}\right)\right)=\frac{\Gamma \left(y_{i}+\frac{1}{\theta }\right)}{\Gamma \left(\frac{1}{\theta }\right)\Gamma \left(y_{i}+1\right)}{\left(\frac{1}{1+\theta \mu_{i}}\right)}^{\frac{1}{\theta }}{\left(\frac{\theta \mu_{i}}{1+\theta \mu_{i}}\right)}^{y_{i}} \end{array}$$

with variance function $Var\left(Y_{i}\right)=\mu_{i}+\theta \mu_{i}^{2}$.

### Best model selection

Selecting the best-fitting model involves balancing model accuracy with parsimony. One of the most widely used criteria for model selection is the Akaike Information Criterion (AIC), which is defined as Eq. (17).

(17) $$\begin{array}{c} AIC=-2\ln\left(L\right)+2k \end{array}$$

where L denotes the maximum likelihood of the fitted model and k is the number of estimated parameters [26]. Models with smaller AIC values are preferred, as they indicate better goodness-of-fit while penalizing unnecessary model complexity.

In the analysis of spatial count data, AIC is particularly useful for comparing baseline models such as Poisson regression and Negative Binomial regression against spatially adaptive models like GWNBR. A substantial reduction in AIC when moving from global models to GWNBR provides evidence that accounting for both overdispersion and spatial heterogeneity leads to a superior model representation [27].

### Computational implementation

All analyses were conducted using R software version 4.3.3. The implementation relied entirely on open-source R packages to ensure transparency and reproducibility. The following R packages and versions were used: MASS (v7.3–60) for Poisson and Negative Binomial regression, car (v3.1–2) for multicollinearity testing using the Variance Inflation Factor (VIF), lmtest (v0.9–40) for the Breusch–Pagan heteroscedasticity test, ape (v5.7–1) for Moran’s I spatial autocorrelation analysis, spgwr (v0.6–36) for kernel-based spatial weighting and bandwidth selection, fields (v14.1) for Euclidean distance computation, zoo (v1.8–12) for auxiliary data handling, and Matrix (v1.6–5) for numerical matrix operations. Matrix operations and numerical inversion were performed using base R and the Matrix package.

Poisson regression was estimated using the glm() function with the poisson family, while Negative Binomial regression was fitted using the glm.nb() function from the MASS package. Spatial dependence was tested using the Moran.I() function, and spatial heterogeneity was assessed via the bptest() function. The Geographically Weighted Negative Binomial Regression (GWNBR) model was estimated using an iterative Newton-Raphson optimization algorithm, implemented through user-defined functions to obtain local parameter estimates and corresponding test statistics at each spatial location. Initial parameter values were obtained from the global Negative Binomial regression estimates to ensure stable convergence across locations. At each spatial location, the weighted log-likelihood function was maximized through iterative updates of the parameter vector based on the gradient and Hessian matrix of the local likelihood. Convergence was determined using a tolerance threshold of $10^{-8}$, defined as the maximum absolute difference between successive parameter estimates. To maintain numerical stability, the iterative process was terminated when convergence was achieved or when the number of iterations reached a predefined maximum of 20 iterations. The Hessian matrix obtained from the final iteration was subsequently used to compute local standard errors and Z-statistics for statistical inference of the estimated parameters.

Four kernel specifications were evaluated, namely adaptive bisquare, adaptive Gaussian, fixed bisquare, and fixed Gaussian kernels. Optimal bandwidths were selected using leave-one-out Cross-Validation (CV) implemented via the ggwr.sel() function. The kernel with the minimum CV value, namely the adaptive bisquare kernel, was selected for the final GWNBR model. In the adaptive specification, bandwidths were allowed to vary across locations to accommodate spatial heterogeneity. Data preprocessing included checking for missing values and consistency verification. Geographic coordinates (longitude and latitude) were extracted for each province and used to compute Euclidean distance matrices between observation locations. The spatial weight matrix was constructed using a distance-based kernel weighting scheme derived from Euclidean distances, rather than a contiguity-based adjacency structure. No polygon shapefiles were required, as the analysis relied exclusively on coordinate-based distance weighting. All spatial computations were conducted using geographic coordinates expressed in decimal degrees.

To ensure reproducibility of the proposed methodology, the following R code snippets illustrate the core functions used at each main stage of the analysis:

**Table:** 

# Poisson Regression pois <- glm(Y ∼ predictors, family = poisson, data = data) # Negative Binomial Regression nb <- glm.nb(Y ∼ predictors, data = data) # Moran's I Test (Spatial Dependence) MI <- Moran.I(data$Y, W) # Breusch-Pagan Test (Spatial Heterogeneity) bptest(nb) # Bandwidth Selection using Adaptive Bisquare Kernel bw <- ggwr.sel(Y ∼ predictors, data = data, coords = cbind(data$Long, data$Lat), adapt = TRUE, gweight = gwr.bisquare) # Geographically Weighted Negative Binomial Regression (custom function) gwnbr <- function(x, y, W, theta) { # x: design matrix (including intercept) # y: response variable # W: spatial weight matrix # theta: dispersion parameter from global NBR # Local parameter estimation at each location using spatial weights and Newton-Raphson optimization return(list( coefficients = …, # local parameter estimates z_values = … # local Z-statistics )) } gwnbr_result <- gwnbr(x, y, W, nb$theta)

The use of open-source software, explicit package versions, and representative code snippets allows the analytical workflow to be replicated by other researchers using the same dataset and methodological framework. Detailed numerical optimization steps for the Newton-Raphson algorithm are described conceptually in the methodology and implemented programmatically within the analysis.

## Method validation

### Real data application

The methodological framework of Geographically Weighted Negative Binomial Regression (GWNBR) described in the previous section is validated through a real data application. The analysis is conducted using provincial-level data obtained from the Central Bureau of Statistics (BPS) and the Ministry of Health of Indonesia for the year 2023. The dataset consists of 34 provinces, which serve as the observational units. The response variable is defined as Number of HIV Cases (y), while the explanatory variables include the Percentage of Poor Population $\left(x_{1}\right)$, Number of Village-Status Areas $\left(x_{2}\right)$, High School Graduates $\left(x_{3}\right)$, and Per Capita Expenditure $\left(x_{4}\right)$. The variables are measured in their original units, where x_1_ is expressed in percentage ( %), x_2_ and x_3_ represent counts, and x_4_ is measured in monetary units (Indonesian Rupiah) ensuring that the model reflects real socioeconomic magnitudes without prior standardization. This dataset is selected because it represents spatially distributed count data with strong socioeconomic linkages, providing an appropriate setting for validating the capability of GWNBR to simultaneously address overdispersion and spatial heterogeneity. The inclusion of poverty and education indicators reflects socioeconomic disparities across provinces, while the number of rural villages and per capita expenditure capture structural and economic dimensions influencing regional development. Beyond its methodological relevance, this application is also aligned with the Sustainable Development Goals (SDGs). Specifically, the analysis relates to SDG 1 (No Poverty), SDG 3 (Good Health and Well-Being), and SDG 4 (Quality Education), which emphasize the interconnectedness of poverty reduction, improved health outcomes, and equitable access to education.

### Multicollinearity test

Before doing the analysis using the Poisson Regression, Negative Binomial Regression, and Geographically Weighted Negative Binomial Regression (GWNBR) methods, the dataset was examined for multicollinearity to assess the presence of high correlations among explanatory variables.. Multicollinearity can be examined through the VIF value. If the VIF value is greater than 10, it can be concluded that there is multicollinearity. Table 1 shows the VIF values between the explanatory variables.

**Table 1:** Multicollinearity test results.

Variable	VIF	Result
Percentage of Poor Population $\left(x_{1}\right)$	1.6732	No multicollinearity
Number of Village-Status Areas $\left(x_{2}\right)$	1.0924	No multicollinearity
High School Graduates $\left(x_{3}\right)$	1.5559	No multicollinearity
Per Capita Expenditure $\left(x_{4}\right)$	2.2298	No multicollinearity

### Poisson regression modeling

The data on the number of HIV cases is assumed to be poisson distributed because it is count data. The following Table 2 is an estimate of the poisson regression model parameters.

**Table 2:** Parameter estimation and significance testing for the poisson regression model.

Parameter	Coefficient	SE	Z-value	P-value	Result
${\hat{\beta }}_{0}$	3.065	0.039150	78.305	0.0000	Significant
${\hat{\beta }}_{1}$	0.01151	0.001518	7.582	0.0000	Significant
${\hat{\beta }}_{2}$	0.00031	0.000002	179.45	0.0000	Significant
${\hat{\beta }}_{3}$	0.00263	0.000623	4.217	0.0000	Significant
${\hat{\beta }}_{4}$	0.00027	0.000003	95.73	0.0000	Significant
Deviance: 24,852					
df: 29					
AIC: 25,151					

Based on the Table 2, the following poisson regression model was obtained:

$$\ln\left(\hat{\mu }\right)=3.065+0.01151\;x_{1}+0.00031\;x_{2}+0.00263\;x_{3}+0.00027\;x_{4}$$

After the model was developed, a simultaneous significance test of the parameters in the poisson regression model was conducted to determine whether the explanatory variables collectively have an effect on the response variable. The hypotheses used are as follows.

$$H_{0}:\beta_{1}=\beta_{2}=\ldots =\beta_{4}=0$$

$H_{1}:$At least one of $\beta_{k}\neq 0$; *k* = 1,2,3,4

Based on the test results with a 5 % significance level, the chi-square critical value is $\chi_{0.05;4}^{2}=\;9.487729$. Therefore, the decision is to reject $H_{0}$ because the value of $D\left(\hat{\beta }\right)=24,852>\chi_{\left(0.05;4\right)}^{2}=9.487729$. This means that at least one explanatory variable has a significant effect on the response variable. Therefore, it is need to continue with partial testing with the following hypothesis:

$$H_{0}:\;\beta_{k}=0$$

$H_{1}:\;\beta_{k}\neq 0$; *k* = 1,2,3,4

Using a 5 % significance level, the critical value is$\;Z_{0.025}=1.96$. Based on Table 2, the absolute values of the Z-values for all parameters exceed $Z_{0.025}$. Therefore, it can be concluded that all explanatory variables used in modeling HIV have a significant effect on the number of HIV cases in Indonesia.

Before applying the Poisson regression model, it is important to ensure that the fundamental assumptions of the model are satisfied, particularly the equidispersion condition, where the conditional mean equals the conditional variance. A key diagnostic is the assessment of overdispersion, since violation of this assumption may render the Poisson model inappropriate. Overdispersion was evaluated using the ratio between the deviance and its corresponding degrees of freedom. A ratio close to one indicates that the equidispersion assumption holds, whereas a ratio substantially greater than one indicates excess variability relative to the Poisson distribution.

For the HIV case data, the model deviance value is 24,852 with 29 degrees of freedom, resulting in a deviance-to-degrees-of-freedom ratio of 856.97, which is far greater than unity. This diagnostic compares the observed variability with that expected under the Poisson assumption. A commonly accepted rule-of-thumb suggests that ratios substantially greater than one (for example, exceeding 1.5 or approaching 2) indicate the presence of overdispersion. The extremely large ratio obtained in this study therefore indicates severe overdispersion in the count data. This overdispersion also explains the unusually large Z-values observed in Table 2. Under the Poisson model, the variance is constrained to equal the mean, which can lead to underestimated standard errors when the data are overdispersed. As a consequence, the resulting Z-values become artificially inflated. Therefore, the large Z-values observed in the Poisson model reflect model misspecification rather than computational error. Based on this diagnostic, the Poisson regression model is considered unsuitable because it may produce biased standard errors and unreliable inference. Therefore, both models were specified under the same systematic component and log-link function, differing only in the variance structure where the Negative Binomial model introduces an additional dispersion parameter to relax the equidispersion assumption.

### Negative binomial regression modeling

Negative binomial regression can be used to address cases of overdispersion that occur in Poisson regression. In modeling using negative binomial regression, there is an initial parameter (θ) that serves to minimize the dispersion parameter, so that it can handle cases of overdispersion.

Based on the Table 3, the following negative binomial regression model was obtained:

$$\ln\left(\hat{\mu }\right)=1.949+0.06453\;x_{1}+0.00035\;x_{2}+0.01687\;x_{3}+0.00026\;x_{4}$$

**Table 3:** Parameter estimation and significance testing for the negative binomial regression model.

Parameter	Coefficient	SE	Z-value	P-value	Result
${\hat{\beta }}_{0}$	1.949	1.16	1.68	0.09305	Not Significant
${\hat{\beta }}_{1}$	0.06453	0.03314	1.947	0.05154	Not Significant
${\hat{\beta }}_{2}$	0.00035	0.00007	5.266	0.0000	Significant
${\hat{\beta }}_{3}$	0.01687	0.01884	0.895	0.37066	Not Significant
${\hat{\beta }}_{4}$	0.00026	0.00009	2.955	0.00313	Significant
Deviance: 37.167					
df: 29					
AIC: 551.13					

Based on this model, it can be interpreted that for every 1 percent increase in the proportion of the poor population $\left(x_{1}\right)$ the number of HIV cases increases by exp(0.06453)=1.0667 cases, assuming that other variables remain constant. Compared with the Poisson model, the Z-values in Table 3 fall within a reasonable range. This occurs because the Negative Binomial model incorporates a dispersion parameter that adjusts the variance structure of the model, thereby correcting the underestimated standard errors observed under the Poisson assumption. Consequently, statistical inference based on the Negative Binomial model becomes more reliable for overdispersed count data.

After the model was developed, a simultaneous significance test of the parameters in the negative binomial regression model was conducted to determine whether the explanatory variables collectively have an effect on the response variable. The hypotheses used are as follows.

$$H_{0}:\beta_{1}=\beta_{2}=\ldots =\beta_{4}=0$$

$H_{1}:$At least one of $\beta_{k}\neq 0$; *k* = 1,2,3,4

Based on the test results with a 5 % significance level, the critical value of $\chi_{0.05;4}^{2}=9.487729$. Therefore, the decision is to reject $H_{0}$ because $D\left(\hat{\beta }\right)=37.167>\chi_{\left(0.05;4\right)}^{2}=9.487729$. This means that at least one explanatory variable significantly influences the response variable. Hence, a partial (individual) test needs to be conducted with the following hypotheses:

$$H_{0}:\;\beta_{k}=0$$

$H_{1}:\;\beta_{k}\neq 0$; *k* = 1,2,3,4

Using a significance level of 5 %, the critical value is $Z_{0.025}=1.96$. Referring to Table 3, the absolute values of the Z-values indicate that two explanatory variables are significant, namely the number of village-status areas $\left(x_{2}\right)$ and per capita expenditure $\left(x_{4}\right)$. Comparing the ratio between the deviance and degrees of freedom, the negative binomial regression has a deviance value of 37.167 with 29 degrees of freedom, resulting in a ratio of 1.282. This ratio is closer to one and smaller than that obtained in the poisson regression, indicating that the negative binomial regression model can address the overdispersion problem present in the poisson regression model.

### Spatial diagnostic testing

Spatial aspects were examined through two main tests:

#### Spatial dependence test

Moran's I statistic was used to identify spatial dependence across regions. The test produced a value of $Z_{I}=3.804898$, which exceeds the critical value $Z_{0.025}=1.96$. Therefore, $H_{0}$ is rejected, indicating a significant spatial dependence in the data.

#### Spatial heterogeneity test

The Breusch–Pagan test was applied to assess variance differences across observation units. The resulting statistic was BP=13.915, which is greater than the critical value $\chi_{\left(0.05;4\right)}^{2}=9.487729$. Thus, $H_{0}$ is rejected, suggesting the presence of spatial heterogeneity.

Since both spatial assumptions are fulfilled, the next step is to conduct an analysis that considers spatial effects, namely through the application of Geographically Weighted Negative Binomial Regression (GWNBR).

### Geographically weighted negative binomial regression modeling

Modeling using the geographically weighted negative binomial regression (GWNBR) method was applied using spatial weights, represented by a matrix of kernel functions. The initial stage involves calculating the euclidean distance between the observation locations. The optimal bandwidth and best weights are then determined on the basis of the minimum cross-validation (CV) value. The selected weights are used to estimate the parameters at each observation point. Details of the CV value calculations for each type of weight are shown in Table 4.

**Table 4:** CV values for each kernel function.

Kernel Function	Bandwidth	CV
Adaptive Bisquare	0.51562	61,908,610
Adaptive Gaussian	0.12833	71,315,617
Fixed Bisquare	11.45506	61,913,862
Fixed Gaussian	4.064267	63,930,296

Based on the results in Table 4, the criterion for selecting the kernel function is the one with the minimum cross-validation value, so the adaptive bisquare kernel function is used to model the number of HIV cases in Indonesia.

### Parameter estimation testing in the GWNBR model

#### Model fit test of GWNBR

This test was conducted to evaluate the suitability of the spatial model compared to the global model. The comparison between the global Negative Binomial Regression (NBR) model and the Geographically Weighted Negative Binomial Regression (GWNBR) model was performed using an F-type statistic based on the ratio of deviance per degree of freedom. The test statistic is constructed by comparing the residual deviance of the global model $D_{global}$ with that of the spatially weighted model $D_{spatial}$, standardized by their respective degrees of freedom. The F-type statistic can be expressed as Eq. (18).

(18) $$\begin{array}{c} F=\frac{D_{global}/df_{1}}{D_{spatial}/df_{2}} \end{array}$$

where $df_{1}$ represents the residual degrees of freedom associated with the global Negative Binomial model and $df_{2}$ represents the effective residual degrees of freedom associated with the GWNBR model. Under the null hypothesis that spatial variation does not significantly improve the model fit, the statistic approximately follows an F distribution. In geographically weighted models, the degrees of freedom are not defined using the conventional global formulation $df_{1}=n-p$. Instead, degrees of freedom are derived from the trace of the spatial hat matrix S. Specifically, the effective residual degrees of freedom can be written as $df_{2}=n-tr\left(S\right)$, while $df_{1}$ follows the standard formulation from the global model. Rejection of the null hypothesis therefore indicates that the spatially varying GWNBR model provides a statistically significant improvement over the global NBR model, consistent with the geographically weighted modeling framework [8].

$H_{0}:\beta_{k}\left(u_{i},v_{i}\right)=\beta_{k}$ (the GWNBR model does not improve the negative binomial regression model)

$H_{1}:\beta_{k}\left(u_{i},v_{i}\right)\neq \beta_{k}$ (the GWNBR model provides a better fit than the negative binomial regression model)

The test results using R software with a significance level of 5 % obtained an F-test statistic value of F=2.3003. The critical region for rejecting $H_{0}$ is if the test statistic $F\;>\;F_{0.05;29;28}$. Thus, the decision is to reject $H_{0}$ because $F=\;2.3003>\;F_{\left(0.05;29;28\right)}=1.8752$. It can be concluded that the GWNBR model is better used than the negative binomial regression model in modeling the number of HIV cases in Indonesia.

#### Simultaneous test of the GWNBR model

Simultaneous testing of the GWNBR model parameters was carried out to determine the significance of the model parameters $\beta_{k}\left(u_{i},v_{i}\right)$ on the response variable. The test was performed using the Maximum Likelihood Ratio Test (MLRT), which compares the deviance of the full model containing all explanatory variables with the deviance of the restricted model. The test statistic is defined as the difference in deviance between the two models and asymptotically follows a chi-square distribution. The degrees of freedom of the test correspond to the number of parameters simultaneously tested, which in this study equals the number of explanatory variables included in the model.

$$H_{0}:\beta_{1}\left(u_{i},v_{i}\right)=\beta_{2}\left(u_{i},v_{i}\right)=\beta_{3}\left(u_{i},v_{i}\right)=\beta_{4}\left(u_{i},v_{i}\right)$$

$H_{1}$: at least one $\beta_{k}\left(u_{i},v_{i}\right)\neq 0$; *k* = 1,2,3,4; *i* = 1,2, …,34

The test statistic $D(\hat{\beta })$ represents the difference in deviance between the restricted model and the full model, which measures the improvement in model fit when the explanatory variables are included in the GWNBR model. The test results using R software with a significance level of 5 %, obtained the value $D(\hat{\beta })=\;8,304.335$. The degrees of freedom for the test are equal to the number of explanatory variables tested simultaneously (df = 4). The critical region for rejecting $H_{0}$ is if $D\left(\hat{\beta }\right)>\chi_{\left(0.05.4\right)}^{2}$. Thus, the decision is to reject $H_{0}$ because $D\left(\hat{\beta }\right)\;=\;8,304.335\;>\;\chi_{\left(0.05.4\right)}^{2}\;=\;9.487729.$ It can be concluded that there is at least one GWNBR model parameter that has a significant influence on the resulting model.

Local parameters at each location were estimated by maximizing the geographically weighted log-likelihood using an iterative Newton-Raphson scheme. Global negative binomial estimates were used as starting values to ensure numerical stability. Convergence was declared when the relative change in the log-likelihood and parameter vector was below $10^{-8}$. The dispersion parameter $\theta (u_{i},v_{i})$ was estimated locally at each location together with the regression coefficients using profile likelihood, allowing spatial variation in overdispersion.

#### Partial test of the GWNBR model

Partial testing of the GWNBR model parameters is used to identify which parameters are significant to the response variable at each observation location. The hypotheses used are as follows.

$$H_{0}:\beta_{k}\left(u_{i},v_{i}\right)=0;k=1,2,3,4;i=1,2,\ldots ,34$$

$$H_{1}:\beta_{k}\left(u_{i},v_{i}\right)\neq 0;k=1,2,3,4;i=1,2,\ldots ,34$$

Based on the test results, different values of Z-value were obtained at each location. The significant variable influencing the number of HIV cases in all provinces was variable Per Capita Expenditure $\left(x_{4}\right)$.

As an example of partial parameter testing, the province of DKI Jakarta was selected. Thus, the hypothesis can be written as follows.

$$H_{0}:\beta_{k}\left(u_{11},v_{11}\right)=0$$

$$H_{1}:\beta_{k}\left(u_{11},v_{11}\right)\neq 0$$

The significance level used is 5 % with a critical region to reject $H_{0}$ if the value |Z| > 1.96. The results of the DKI Jakarta parameter testing are presented in Table 5.

**Table 5:** Estimation results of GWNBR parameters in DKI Jakarta.

Parameter	Coefficient	Z-values	Result
$\beta_{0}$	1.734573	106,860	Significant
$\beta_{1}$	1.943409	−3.30808	Significant
$\beta_{2}$	−0.07275	9814.021	Significant
$\beta_{3}$	0.000573	4.053285	Significant
$\beta_{4}$	0.105684	48.10864	Significant
θ	0.000015	-	

Based on Table 5, the absolute Z-values for parameters $\beta_{1}$, $\beta_{2}$, $\beta_{3}$, and $\beta_{4}$ exceed the critical value of 1.96, indicating that all explanatory variables significantly affect the number of HIV cases in DKI Jakarta at the 5 % significance level. Several parameters exhibit very large Z-statistics. This occurs because the geographically weighted estimation produces location-specific standard errors derived from the inverse Hessian matrix of the local likelihood function. In locations where the spatially weighted observations provide strong local information, the estimated standard errors may become very small, resulting in large absolute Z-values. The heterogeneity of Z-values, including large magnitudes or differing signs, reflects spatial variation in local information and weighting. The dispersion parameter estimated for DKI Jakarta is very small (near-zero), indicating that the local variance structure at this location approaches the Poisson assumption. This observation does not contradict the earlier detection of global overdispersion. The overdispersion identified in the global Poisson model may partly arise from spatial heterogeneity across provinces. Because the GWNBR model estimates parameters locally using spatially weighted observations, the variance structure can differ across locations. Consequently, some regions may exhibit variance patterns that are close to equidispersion, resulting in a near-zero dispersion parameter. This finding therefore reflects spatial variation in dispersion rather than inconsistency with the earlier global diagnostics.

The model for the number of HIV cases in DKI Jakarta can be written as follows:

$$\hat{\mu }=\exp\left(1.7346+1.9434X_{1}-0.0728X_{2}+0.0006X_{3}+0.1057X_{4}\right)$$

Because the explanatory variables are measured on different scales, the coefficients are interpreted relative to a one-unit change in each variable according to its original measurement scale. Therefore, the multiplicative effects obtained from exp(β) reflect proportional changes in the expected number of HIV cases within each variable rather than direct magnitude comparison across variables. Based on the model formed for DKI Jakarta, it can be interpreted that for every 1 unit increase in the percentage of poor population $\left(x_{1}\right)$ assuming other explanatory variables remain constant, the number of HIV cases in DKI Jakarta will increase by exp(1.9434)=6.9825. Furthermore, for every 1 unit increase in number of village-status areas $\left(x_{2}\right)$ assuming other explanatory variables remain constant, the number of HIV cases will decrease by exp(−0.0728)=0.930. Likewise, a 1 unit increase in the number of senior high school graduates $\left(x_{3}\right)$ assuming other explanatory variables remain constant, will increase the number of HIV cases by exp(0.0006)=1.0006. Finally, a 1 unit increase in per capita expenditure $\left(x_{4}\right)$ assuming other explanatory variables remain constant, will increase the number of HIV cases by exp(0.1057)=1.1115.

### Selection of the best model

The best model refers to a model that has the capacity to explain the relationship between explanatory variables and response variables. The selection of the best model is done using the minimum Akaike Criterion Information (AIC). A comparison of the AIC values for each model is presented in Table 6 as follows.

**Table 6:** Comparison of AIC values for each regression model.

Model	AIC
Poisson Regression	25,151
Negative Binomial Regression	551.13
Geographically Weighted Negative Binomial Regression	488.71

Based on Table 6, the AIC value of the GWNBR model is 488.71. which is lower than the AIC values of both the poisson regression model and the negative binomial regression model. Therefore. it can be concluded that the GWNBR model is the optimal model for modeling the number of HIV cases in Indonesia. The AIC values obtained for the Poisson, NBR, and GWNBR models exhibit substantial differences in magnitude. This discrepancy primarily reflects differences in model specification and likelihood structure. The Poisson model assumes equidispersion, which is clearly violated in the present data as indicated by the severe overdispersion diagnostic. Consequently, the Poisson model produces a much poorer likelihood fit, resulting in a substantially larger AIC value. The Negative Binomial regression model improves the variance specification by introducing a dispersion parameter, which leads to a considerable reduction in the AIC value. The GWNBR model further extends this framework by allowing model parameters to vary spatially through geographically weighted estimation. By capturing spatial heterogeneity in the relationships between predictors and HIV cases, the GWNBR model achieves a better fit to the localized data structure, which results in a further reduction in the AIC value. Therefore, the large difference in AIC magnitude reflects the progressive improvement in model specification from a global equidispersed model to a spatially localized count model, rather than a scaling inconsistency.

**Fig. 1:**
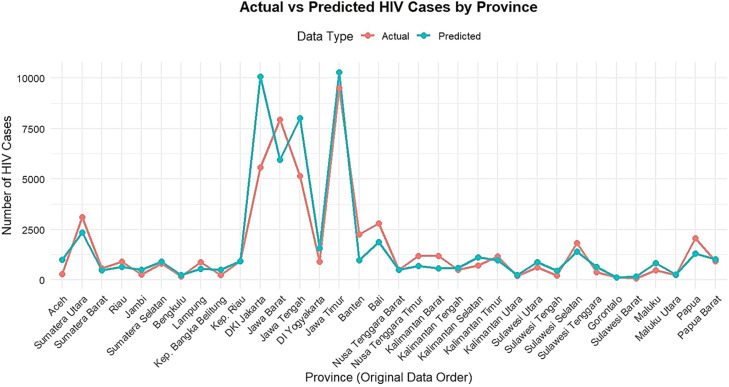
Comparison of actual values (y) and predicted values $\left(\hat{y}\right)$.

The results of the comparison between the estimated number of HIV cases ($\hat{y}$) and the actual number (y) are presented in Fig. 1 as follows.

Based on Fig. 1, the predicted values of HIV cases are fairly close to the actual values, especially following the general pattern of increase and decrease across various observation locations. However, there are noticeable differences in areas with a very high numbers of cases, indicating that the model is not yet fully accurate in predicting extreme cases.

The spatially varying relationships identified by the GWNBR model provide important implications for public health policy. The results indicate that the determinants of HIV cases differ across provinces, suggesting that uniform national interventions may not be fully effective. In several provinces, education appears to play a stronger protective role, implying that strengthening educational access and HIV awareness programs may help reduce transmission risk. In contrast, poverty and rural characteristics remain dominant contributors in other regions, highlighting the importance of targeted socioeconomic and community-based interventions. These findings emphasize the need for localized, evidence-based public health strategies, where prevention and control programs are tailored according to province-specific risk profiles rather than relying solely on national averages. Therefore, spatial modeling such as GWNBR can support policymakers in identifying high-risk provinces, optimizing resource allocation, and designing geographically targeted HIV prevention strategies.

## Limitations

This study has several limitations that should be considered when interpreting the results. First, the analysis is based on provincial-level aggregated data, which may mask within-province variability and local heterogeneity. Consequently, the estimated relationships represent regional patterns rather than individual-level dynamics. Second, the data used in this study are cross-sectional, capturing spatial variation at a single point in time. Therefore, the analysis cannot account for temporal dynamics or changes in HIV incidence and socioeconomic factors over time. Third, the performance of the GWNBR model may be sensitive to bandwidth selection and kernel specification, which determine the spatial weighting structure of the local estimation. Although cross-validation was used to determine the optimal bandwidth, different kernel choices or bandwidth selection criteria may produce slightly different local parameter estimates. Fourth, spatial weighting based on geographic distance may introduce edge effects and may depend on coordinate projection and distance measurement. In regions located near the boundary of the study area, fewer neighboring observations may influence the stability of local parameter estimates. Finally, the stability of geographically weighted models can also be affected by sample size and local data density, particularly in regions with relatively limited observations. Future research may extend this work by incorporating panel data, alternative spatial weighting schemes, or multiscale geographically weighted approaches to further explore spatial heterogeneity in HIV incidence.

## Ethics statements

The dataset was obtained from the Central Bureau of Statistics and the Ministry of Health of Indonesia. The sources include the 2023 Provincial Publication in Figures, the 2023 Human Development Index (HDI) Report, and official records of HIV case counts published in the Quarterly HIV/AIDS and Sexually Transmitted Infection Report by the Ministry of Health. All statistical publications are publicly accessible through the official BPS repository at https://www.bps.go.id/id/publication and the Ministry of Health portal at https://siha.kemkes.go.id.

## CRediT authorship contribution statement

**Toha Saifudin:** Validation, Supervision. **Nur Chamidah:** Writing – review & editing. **Nur Azizah:** Conceptualization, Methodology, Software, Formal analysis. **Fayza Shafira Renianti:** Writing – original draft, Resources. **Nashwa Carista:** Visualization, Investigation. **Irsyad Yoga Adyatma:** Writing – review & editing. **Izhar Muhammad Tianda:** Software, Data curation.

## Declaration of competing interest

The authors declare that they have no conflicting financial interests or personal relationships that could be considered to influence the work reported in this paper.

## Data Availability

Data will be made available on request.
